# New cytokinin derivatives – their discovery, development and use for micropropagation of endangered tree species

**DOI:** 10.1186/1753-6561-5-S7-O46

**Published:** 2011-09-13

**Authors:** Karel Dolezal, Jana Malá, Pavlína Máchová, Helena Cvrcková, Michal Karady, Ondrej Novák, Lucie Szucova, Jaromir Mikulik, Lukas Spichal, Miroslav Strnad

**Affiliations:** 1 Laboratory of Growth Regulators, Palacký University and Institute of Experimental Botany AS CR, 783 71 Olomouc-Holice, The Czech Republic; 2 Institute of Forest and Game Management, Strnady 136, 252 02 Jílovišt?, The Czech Republic; 3 Centre of the Region Haná for Biotechnological and Agricultural Research, Faculty of Science, Palacký University, Šlechtitel? 11, CZ 783 71 Olomouc, Czech Republic

## 

Elms (*Ulmus* sp.) are highly valuable trees for their great strength, tightly twisted grain, and durability, as well as for their cold and salt tolerance[1]. Wych elm (*Ulmus glabra*, Huds.) is a native species in Europe. In the Czech Republic, it is spread especially in mountain regions. During the years 1970–80, the second epidemic of Dutch elm disease destroyed most elm populations in Europe. The elm trees, which have survived the disease, are capable to maintain a high degree of resistance against the repeated infection. Micropropagation represents promising alternative of their vegetative reproduction.

The wild service tree (*Sorbus torminalis* (L.) Crantz) is a slow-growing tree reaching maximum height of 20–25 m at around 80–100 years. According to the latest data, the species is also disappearing from forests of the Czech Republic and it is considered to be endangered. On the other hand, the wild service tree is rated as one of the most valuable hardwoods [2] with a great potential for wider use in forestry and forest ecology, and also for its importance in the timber industry.

The *in vitro* induction of organogenesis, rooting and acclimatization of above mentioned elm species as well as wild service tree were optimalized and standartized. Widely used BAP is an important and affordable cytokinin routinely utilized for its effective stimulatory properties in micropropagation practice. On the other hand, BAP may negatively influence the growth, rooting and acclimatization processes in some crops [3].It is known that hydroxylated aromatic cytokinins (topolins) are more resistant to cytokinin oxidase (CKX), more stable and in some biological systems active at lower concetrations than the isoprenoid CKs. In addition, *meta* - topolin does not inhibit root formation which is a typical inconvenient effect of high concentrations of BAP. These properties might help to enhance the future productivity of plant tissue culture industry crops [3,4]. The development of other new CK derivatives exhibiting high morphogenetic activity might consequently be of a great practical importance in plant biotechnology.During our recent search for naturally occurring aromatic cytokinins (ARCKs) in plants, another new group of endogenous BAP derivatives –methoxytopolins – exhibiting high biological, especially anti-senescence (and surprisingly also anti-cancer) activity, was discovered. Based on these results, we synthesized several groups of their synthetic analogues that exhibited high activity in three different CK bioassays and showed the ability to activate cytokinin receptors and/or to inhibit CKX. Best compounds so far (6-(3-hydroxybenzylamino)purine (mT) and the 6-(3-methoxybenzylamino)purine-9-ribofuranoside (MeomTR)) were used for retardation of senescence during multiplication stage of micropropagation of above mentioned tree species as well as for rooting support. Subsequently, wide range of endogenous plant hormones (isoprenoid cytokinins, IAA, ethylene) were quantified (using UPLC-MS/MS and/or GC/FID, respectively) and compared in relation to cytokinin exogenously used.

The results about optimal endogenous plant hormone concentrations and their dependence on different exogenous cytokinin used in the cultivation media, may improve *in-vitro* micropropagation efficiency as well as quality of *ex vitro* acclimatized plants of above mentioned elm species, wild service tree and possibly also of other tree species.

This work was supported by the Ministry of Agriculture of the Czech Republic (Mze 0002070202, QI 92A247), Czech Ministry of Education (MSM 6198959216), ED0007/01/01 Centre of the Region Haná for Biotechnological and Agricultural Research.
